# Bortezomib induces AMPK-dependent autophagosome formation uncoupled from apoptosis in drug resistant cells

**DOI:** 10.18632/oncotarget.2590

**Published:** 2014-10-18

**Authors:** Sajjeev Jaganathan, Ehsan Malek, Subrahmanya Vallabhapurapu, Sivakumar Vallabhapurapu, James J. Driscoll

**Affiliations:** ^1^ Department of Internal Medicine, University of Cincinnati College of Medicine, The Vontz Center for Molecular Studies, Cincinnati, OH 45267; ^2^ Division of Hematology and Oncology, University of Cincinnati College of Medicine, Cincinnati, OH 45267; ^3^ Department of Cancer Biology, University of Cincinnati College of Medicine, Cincinnati, OH 45267

**Keywords:** Myeloma, proteasome, drug Resistance, autophagy, apoptosis

## Abstract

The proteasome inhibitor bortezomib is an effective anti-cancer agent for the plasma cell malignancy multiple myeloma but clinical response is hindered by the emergence of drug resistance through unknown mechanisms. Drug sensitive myeloma cells were exposed to bortezomib to generate drug resistant cells that displayed a significant increase in subunits of the energy sensor AMP-activated protein kinase (AMPK). AMPK activity in resistant cells was increased and bortezomib resistant cells contained a ~4-fold greater level of autophagosomes than drug sensitive cells. Real-time measurements indicated that bortezomib reduced the oxygen consumption rate in drug sensitive cells more readily than in resistant cells. Genetic ablation of AMPK activity reduced the bortezomib effect on autophagy. The autophagy-related gene (*Atg*)5 is required for autophagosome formation and enhances cellular susceptibility to apoptotic stimuli. *Atg5* knockout eliminated bortezomib-induced autophagosome formation and reduced susceptibility to bortezomib. Bortezomib treatment of myeloma cells lead to ATG5 cleavage through a calpain-dependent manner while calpain inhibition or a calpain-insensitive *Atg5* mutant promoted bortezomib-resistance. In contrast, AICAR, an AMPK activator, enhanced bortezomib-induced cleavage of ATG5 and increased bortezomib-induced killing. Taken together, the results demonstrate that ATG5 cleavage provokes apoptosis and represents a molecular link between autophagy and apoptosis with therapeutic implications.

## INTRODUCTION

Precisely regulated quality control systems monitor proper folding, assembly and functionality of cellular proteins [1, 2]. Eukaryotic cells have developed efficient strategies to cope with misfolded, denatured or defective proteins. Molecular chaperones refold unfolded or denatured proteins to restore their native conformation, but, if these systems fail, these proteins are rapidly destroyed by the ubiquitin + proteasome system (UPS) [3, 4]. The UPS is a highly complex network that maintains cell viability through the selective turnover of targeted proteins. The proteasome serves as the catalytic core of the UPS to efficiently degrade short-lived and denatured proteins [5]. Bortezomib is a potent and selective proteasome inhibitor that exploits its pivotal biologic role to promote cell death [6, 7]. Bortezomib has improved the response and survival of patients with the plasma cell (PC) malignancy multiple myeloma (MM) [8, 9]. While success of bortezomib has emerged as standard-of-care therapy to catapult the UPS into a position of prominence in cancer biology, significant obstacles remain [10, 11]. Many patients do not respond to proteasome inhibitors and those that do inevitably develop drug resistance. There is an urgent, unmet need to unravel the mechanism(s) of resistance to proteasome inhibitors.

The molecular events that regulate the complex interplay between autophagy and apoptosis as determinants of cell fate under physiologic and pathologic conditions remain poorly understood. Cancer cells display differential aspects of metabolism relative to normal, differentiated cells and face enormous metabolic challenges to meet the energetic and biosynthetic demands of increased proliferation [12, 13]. AMPK is the major cellular energy sensor and master regulator of metabolic homeostasis [14]. AMPK activation is triggered by an increase in the AMP/ATP ratio to promote ATP production, increase catabolism and conserve ATP by switching off anabolic pathways [15]. Pharmacologic modulation of AMPK presents a unique opportunity to reverse cancer-related metabolic abnormalities [16, 17].

Autophagy is an evolutionarily conserved, dynamic process that promotes cellular homeostasis through the catabolism and recycling of intracellular proteins and organelles [18, 19]. Autophagy requires the concerted action of multiple cytoplasmic proteins that generate double membrane-bound structures termed autophagosomes. The resulting double-membrane bound vesicles then fuse with lysosomes to deliver their cytoplasmic cargo for lysosomal disposal. Deregulation of autophagy contributes to numerous maladies such as human cancers neurodegenerative diseases and aging [20–22]. However, validated targets in this pathway are scant and treatment strategies are limited by the lack of effective or specific agents without pharmacologic liabilities. Treatment of transformed cells with chemotherapeutics triggers autophagy as a pro-survival mechanism to remove damaged, oxidized, or aggregated proteins [23, 24]. We reasoned that resistance to proteasome inhibition could be achieved through metabolic adaptation that may not only increase autophagy but also uncouple autophagy from apoptosis. We demonstrate that AMPK triggers bortezomib-induced autophagy linked to the coordinated induction of cell apoptosis. ATG5 is required for autophagy and functions in apoptosis as a dual-functioning effectors. Calpain-mediated cleavage eliminates the capacity of ATG5 to promote autophagy but enhances the pro-apoptotic activity. Deregulation of ATG5 may uncouple these processes and promote drug resistance in cancer cells.

## RESULTS

**Figure 1:**
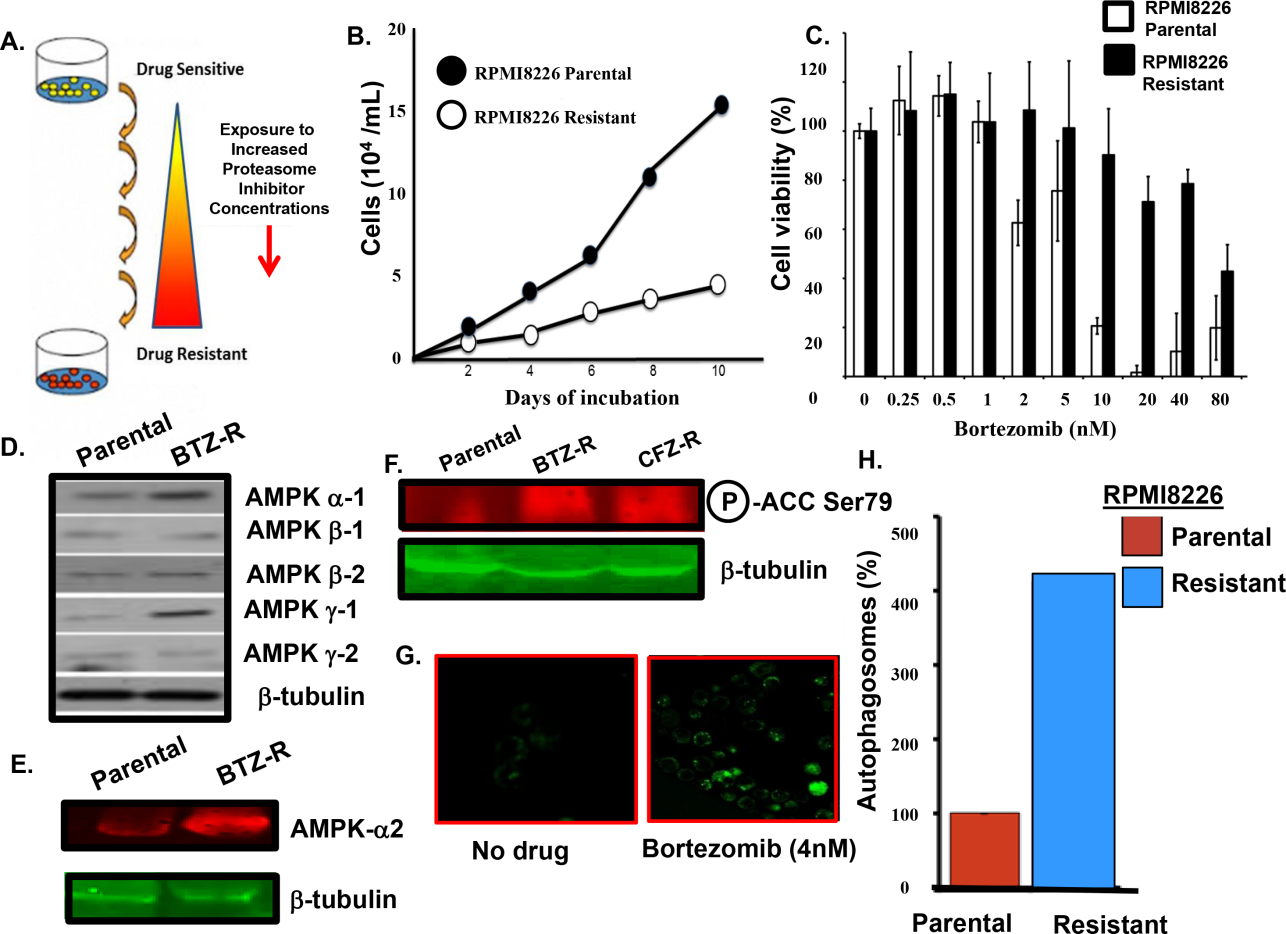
Bortezomib-resistant myeloma cells **(A)** Scheme used to generate bortezomib-resistant cells. **(B)** Growth rate comparison of parental and drug-resistant cells as measured by trypan blue staining. **(C)** Viability of parental and bortezomib-resistant cells after bortezomib addition at the indicated concentrations. Viability was determined using the XTT assay and the values represent the arithmetic mean of triplicate measurements and error bars represent the standard deviation. **(D)** Western blot to compare the level of individual AMPK subunits in the parental and bortezomib-resistant cells. Lysates from parental or resistant cells were probed using antibodies to the AMPK alpha 1/2, beta 1/2 and gamma 1/2 subunits. **(E)** Western blotting to compare the level of AMPK alpha-2 subunit in parental and bortezomib-resistant cells. **(F)** Western blotting to compare the level of phospho-Ser79 ACC in parental and drug-resistant myeloma cells. **(G)** Confocal microscopy to detect autophagosomes. Cells treated with bortezomib, pelleted, incubated with the cyto-ID green detection reagent, applied to a microscope slide and visualized using a Zeiss LSM170 confocal microscope. **(H)** Quantitation of autophagosomes in parental and drug-resistant cells. Cells were incubated with the cyto-ID detection reagent and autophagosomes quantitated by flow cytometry. Shown is the result of triplicate measurements.

*Bortezomib-resistant myeloma cells.* To investigate the molecular mechanism of acquired resistance to bortezomib, the myeloma cell line RPMI8226 was subjected to successively increased concentrations of the proteasome inhibitors bortezomib (Figure 1A). Bortezomib-resistant cells in culture grew more slowly than drug-naïve parental cells and were not as sensitive as parental cells to proteasome inhibitors by XTT assay (Figure 1B, C). AMPK controls nutrient sensing and energy homeostasis and serves an essential role in autophagy induction [26]. Western blotting indicated that the levels of individual AMPK alpha-1, alpha -2 and gamma -1 subunits were increased in the bortezomib-resistant cells (Figure 1D, E). AMPK activity was monitored by western blot using an antibody that specifically recognized phosphorylation of the AMPK substrate acetyl co-A carboxylase (ACC) on serine 79 (Ser79). AMPK activity was much higher in bortezomib-resistant cells compared to the drug-naïve cells (Figure 1F). Resistant cells displayed IC_50_ values for bortezomib-induced cytotoxicity at least >5-fold higher than those obtained for parental (bortezomib-sensitive) cells. We reasoned that bortezomib-mediated inhibition of the UPS has a stimulatory effect on autophagy as a potential compensatory protein clearance mechanism. Cells were also treated with bortezomib, stained with a dye specific for autophagosome detection and examined by confocal microscopy. We observed a substantial increase in green fluorescence within bortezomib-treated cells (Figure 1G). A live cell-based flow cytometry then quantitated the bortezomib effect on autophagosomes in myeloma cells (Figure 1H). The method monitors autophagic flux using a novel dye that selectively labels autophagic vacuoles and eliminates the need for plasmid transfection, drug selection and validation of fluorescent reporters. Importantly, the number of autophagosomes detected in bortezomib-resistant cells was significantly higher than that seen in parental cells.

**Figure 2:**
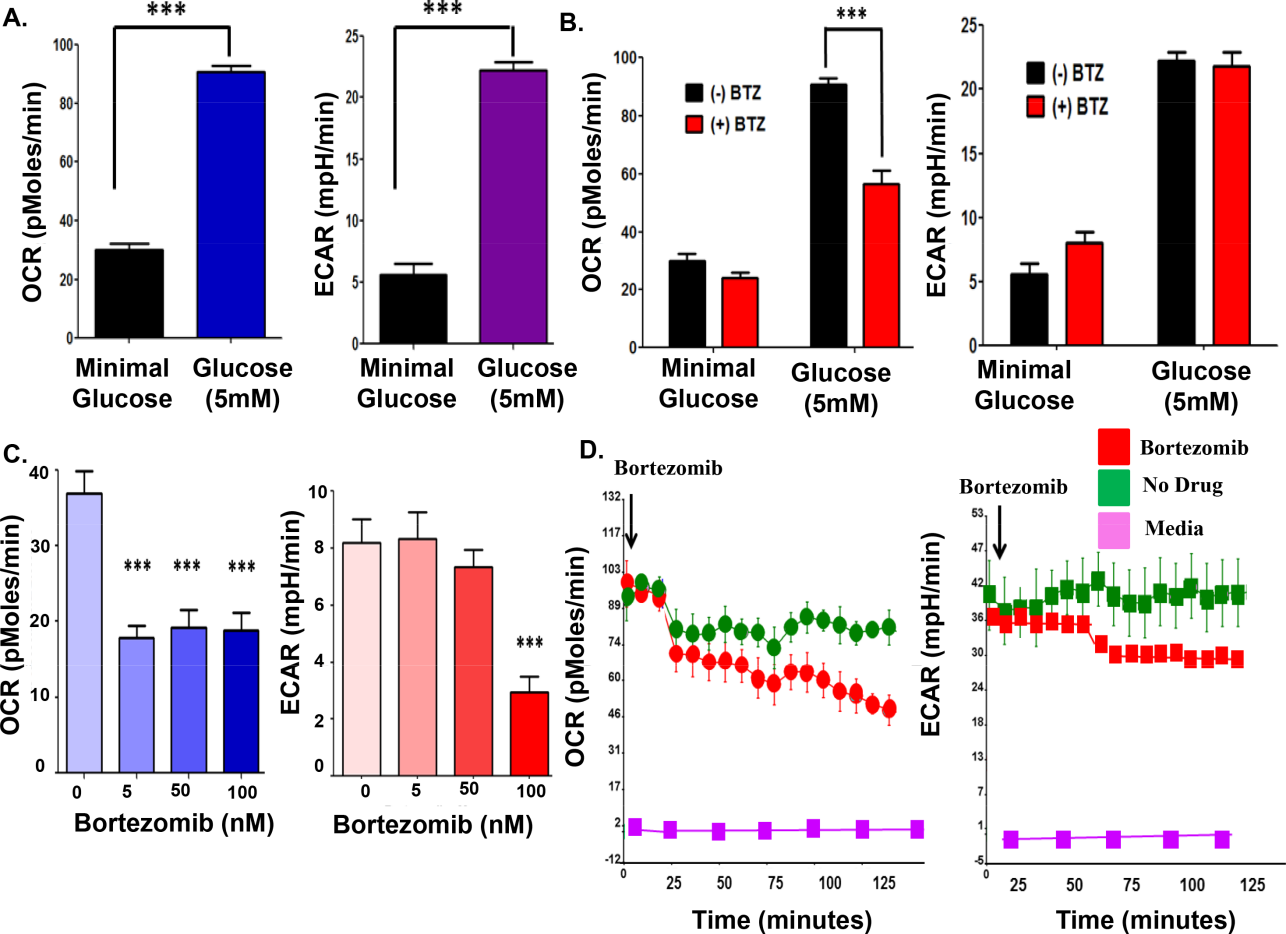
Bortezomib effect on the OCR and ECAR in parental myeloma cells **(A)** Glucose effect on the OCR and ECAR in parental myeloma cells. **(B)** Bortezomib effect on the OCR and ECAR of parental myeloma cells. Cells were grown in minimal or 5 mM glucose for 24 hours prior to measurements. **(C)** Dose-dependent effect of bortezomib on the OCR and ECAR measurements. **(D)** Real-time measurements to determine the effect of bortezomib on OCR and ECAR in parental myeloma cells. Bortezomib was injected directly into wells that contained RPMI8226 cells and recorded over 150 minutes.

*Real-time (RT) measurement of myeloma cell bioenergetics.* Cellular bioenergetics processes contribute to the progression of disease and related to it, oxidative phosphorylation (OXPHOS) and glycolysis have prominent roles in cancer cells. Seahorse flux analyzers allow the measurement of oxygen consumption rates (OCR) as a measure of OXPHOS, and extracellular acidification rates (ECAR) as a measure of lactate production by glycolysis. The system allows the investigator to simultaneously interrogate the two major energy producing pathways of the cell – mitochondrial respiration and glycolysis - in a microplate, in real-time. The measurement of cellular bioenergetics on live cells enables time-resolved analysis and testing of multiple conditions per assay well. Seahorse assays provide increased throughput and use less sample compared to conventional respirometry techniques. Since proteasome inhibition increased AMPK activity, we examined the effect of bortezomib on fundamental metabolic parameters such OCR and ECAR in real-time using a Seahorse analyzer. Glucose (5 mM) addition to cells resulted in a substantial increase in both the basal OCR and ECAR relative to cells grown in minimal glucose-containing media (2 mM) (Figure 2A). The addition of bortezomib reduced the OCR in cells grown in either low or high glucose but the effect was more prominent in the cells grown in high glucose (Figure 2B). ECAR did not significantly change with bortezomib. The effect of bortezomib on OCR was seen at drug concentrations as low as 5 nM (Figure 2C). RT measurements indicated that bortezomib injection into myeloma cells in suspension rapidly reduced the OCR by 60% in 120 minutes (Figure 2D).

**Figure 3:**
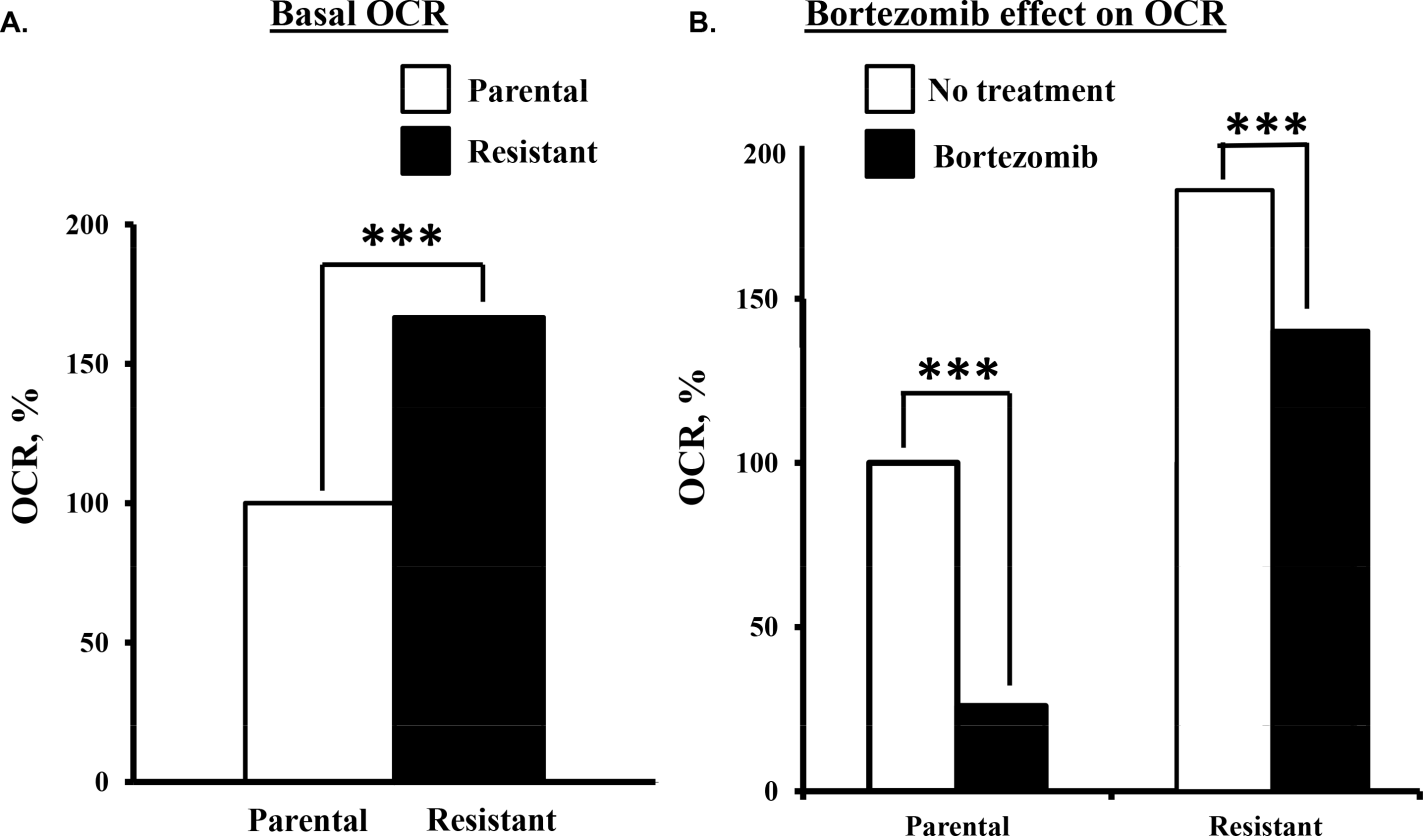
Comparison of the bortezomib effect on parental and bortezomib-resistant cells **(A)** Basal OCR measurements in parental and bortezomib-resistant cells. **(B)** Parental and bortezomib-resistant cells were treated with bortezomib (10 nM) and OCR then measured.

*Bortezomib effect on the metabolic parameters of drug-resistant myeloma cells.* We then determined that the basal OCR was >50% higher in bortezomib-resistant cells than parental cells (Figure 3A). Also, bortezomib-resistant cells were less sensitive to the drug-induced effects on the OCR (Figure 3B). Two hours after bortezomib addition, the OCR in parental cells was reduced by ~75% while in drug-resistant cells the OCR was reduced by only ~25%.

**Figure 4:**
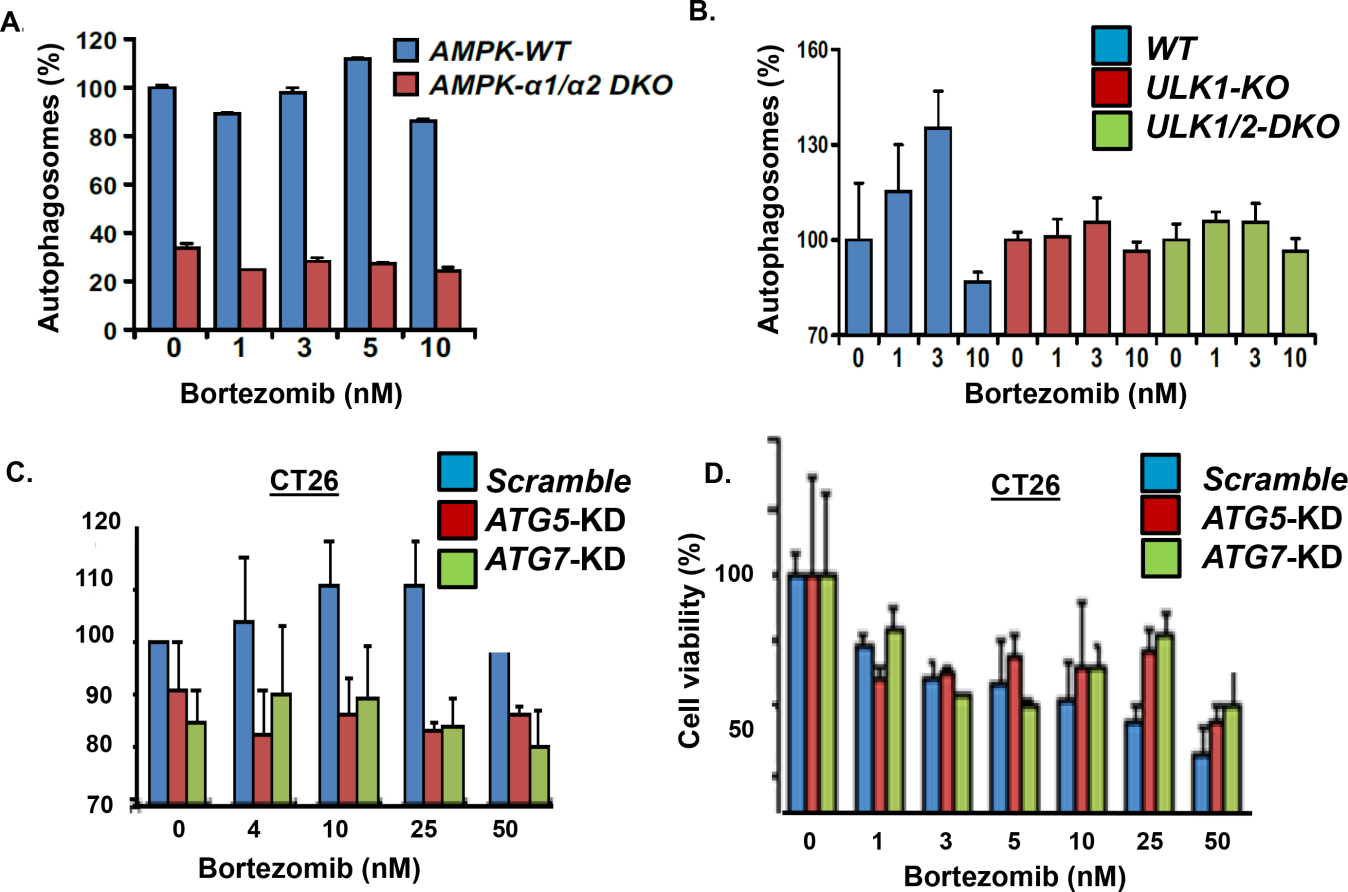
Effect of *AMPK, ULK, ATG5* and *ATG7* genetic knockout **(A)** Effect of *AMPK-alpha1/2* knockout on bortezomib-induced autophagosome formation. *AMPK-WT* and *AMPK-alpha1/2-DKO* MEFs were incubated with bortezomib at indicated concentrations for 18 hours and autophagosomes quantitated using the cytoID kit and flow cytometry. Shown is the average of triplicate measurements. **(B)** Effect of *ULK-1* and *ULK-1/2* knockout on bortezomib-induced autophagosome formation. *ULK-WT* and *ULK-1/2-DKO* MEFs were incubated with bortezomib at indicated concentrations for 18 hours and autophagosomes quantitated using the cyto-ID method. Shown is the average of triplicate measurements. **(C)** CT26 cells were transfected with control siRNA or siRNA to knockdown *Atg5* or *Atg7,* treated with bortezomib and autophagosomes quantitated. **(D)** Effect of *Atg5* or *Atg7* silencing on bortezomib-induced cell death. Cells were treated with bortezomib at indicated concentrations and viability determined. Shown are the results of triplicate measurements. Error bars represent standard deviations.

*Effect of AMPK knockout on bortezomib-induced autophagy.* The results indicated that the protein level and catalytic activity of AMPK was upregulated in bortezomib-resistant cells. Since AMPK activation promotes autophagosome formation, we determined the effect of genetic ablation of the AMPK catalytic subunits on bortezomib-induced autophagosome formation (Figure 4A). The AMPK-α1/α2 catalytic subunits were knocked out (*AMPK-DKO*) in in mouse embryonic fibroblasts (MEFs) and these MEFs were treated with bortezomib as indicated. The results showed that the genetic ablation of the AMPK catalytic subunits significantly reduced the basal level of autophagosomes in *AMPK-DKO* MEFs compared to *AMPK-WT* MEFs. In addition, the number of autophagosomes detected in *AMPK-WT* MEFs was increased by bortezomib treatment but not in the *AMPK-DKO* MEFs. The lack of a bortezomib effect was seen at concentrations up to 10 nM. The results indicate that even at relatively high drug concentrations, genetic ablation of AMPK eliminated the capacity to form autophagosomes.

*ULK1/ATG1* is a substrate of AMPK activated through AMPK-mediated phosphorylation to promote formation of structural precursors of autophagosomes called autophagophores [27, 28]. In yeast, autophagy results in ATG13 dephosphorylation, association between ATG13 and ATG1 and full activation of ATG1 kinase activity. Mammalian homologues of ATG1, e.g., ULK1/2, form a similar complex with the mammalian ATG13 protein, and are necessary for proper autophagy induction. We determined the effect of bortezomib at increased concentrations on autophagosome formation in *ULK1/2-KO* MEFs relative to *ULK-WT* controls. Knockout of *ULK1* or both *ULK1*/*2* reduced bortezomib-induced autophagosome formation compared to the *ULK-WT* MEFs (Figure 4B). Across a broad concentration range, bortezomib did not induce autophagosomes in either *ULK1-KO* or *ULK1/2-DKO* MEFs to suggest that *ULK1/2*, like *AMPK*, was essential for bortezomib-induced autophagosome formation. Bortezomib treated of either AMPK-WT MEFs, ULK-WT MEFs or CT26 colon carcinoma cells led to a slight increase in the number of autophagosomes. While the effect of bortezomib across a broad concentration range was not dramatic, it consistently trended in the same direction and was observed in multiple cell types. While the effect of bortezomib across a broad concentration range was not dramatic, the effect was consistently observed in multiple cell types and trended in the same direction. It is possible that the cyto-ID-based flow cytometry method detects relatively large differences in autophagosome numbers, e.g., the difference between *AMPK-WT* and *AMPK-DKO* MEFs (Figure 4A), better than the subtle differences seen after bortezomib treatment.

Enforced *Atg5* expression sensitizes tumor cells to chemotherapy *in vitro* and *in vivo* while *Atg5* silencing results in chemotherapeutic resistance [29]. Since ATG5 was shown to enhance susceptibility to apoptotic stimuli, we investigated the effect of *Atg5* silencing on bortezomib-induced autophagosome formation and cell death (Figure 4C, D). CT26 colon carcinoma cells were transfected with siRNA directed against either scrambled, *Atg5* or *Atg7.* Cells were treated with bortezomib and those transfected cells with *Atg5* or *Atg7* siRNA did not form autophagosomes to the same level as cells transfected with scrambled siRNA. Cells transfected with *Atg5* or *Atg7* siRNA were also less sensitive to bortezomib-induced cell death since these cells did not demonstrate a significant increase in autophagosomes across a broad drug concentration range.

**Figure 5:**
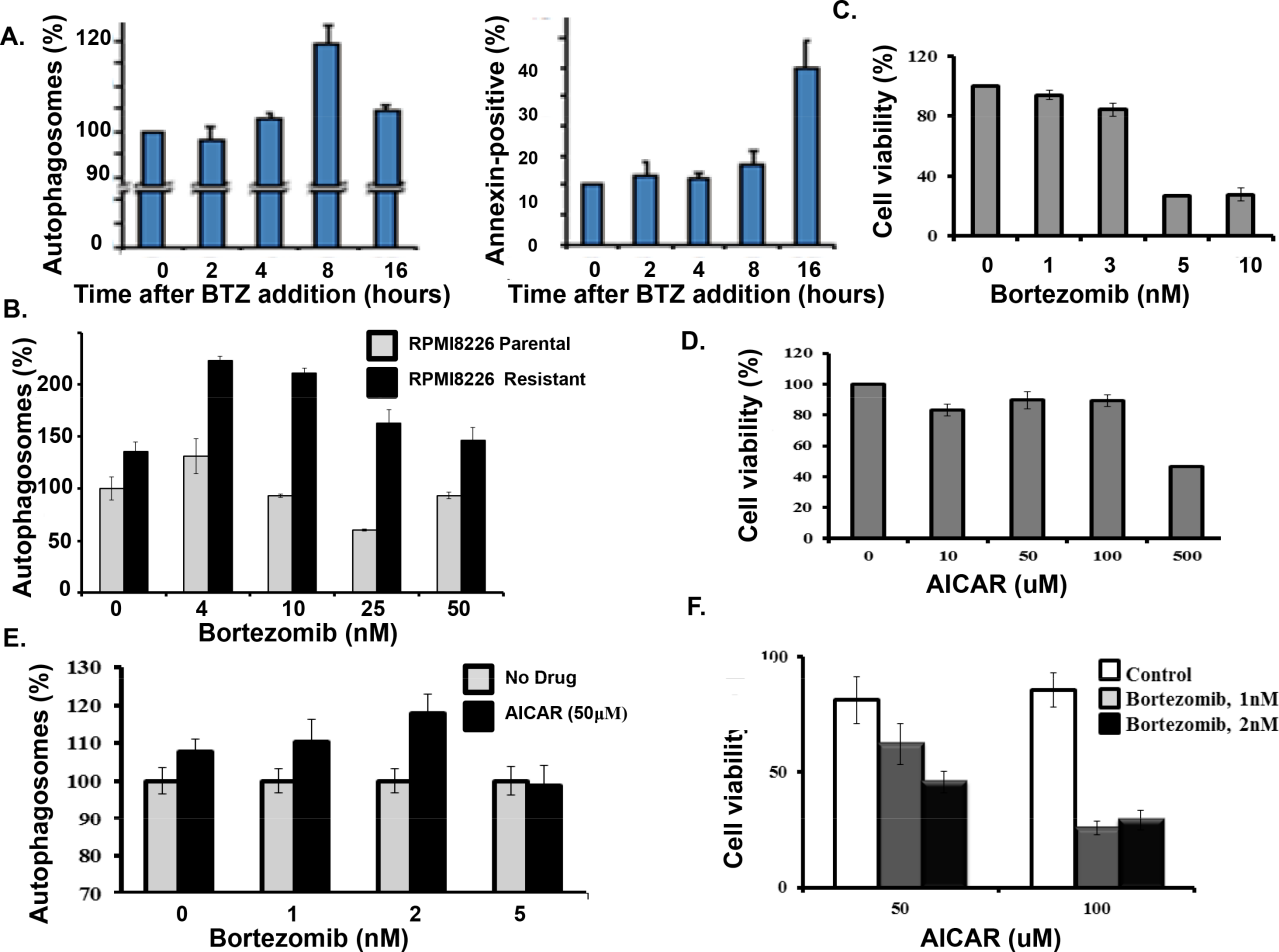
AICAR effect on bortezomib-induced cell death **(A)** Time course of bortezomib effect on autophagosome formation and apoptosis in myeloma cells. Autophagosomes were quantitated using the dye-based cytoID reagent and flow cytometry. The percentage of annexin-positive cells was determined by flow cytometry. Shown are the results of triplicate measurements. Error bars represent the standard deviation. **(B)** Autophagosome formation in drug-sensitive and drug-resistant myeloma cells. **(C)** Effect of bortezomib on myeloma cell viability. Cells were treated with bortezomib at indicated concentrations and viability determined by the XTT assay. Shown are the results of triplicate measurements. Error bars represent the standard deviations. **(D)** AICAR effect on myeloma cell viability. Cells were treated with bortezomib at indicated concentrations and viability determined by the XTT assay. Shown are the results of triplicate measurements. Error bars represent the standard deviations. **(E)** AICAR effect on bortezomib-induced autophagosome formation. **(F)** AICAR effect bortezomib-induced cell death. Cells were treated with bortezomib at indicated concentrations and viability determined by the XTT assay. Shown are the results of triplicate measurements. Error bars represent the standard deviations.

Bortezomib induced the formation of autophagosome at much earlier time points than the drug induced the generation of annexin-positive cells (Figure 5A). Also, bortezomib treatment of the drug-resistant cells led to a significantly greater increase in autophagosomes than in parental cells (Figure 5B). We reasoned that enhanced autophagosome formation in the resistant cells might reflect the uncoupling of autophagy from apoptosis. To further address these findings, myeloma cells were treated with bortezomib at the indicated concentrations. Bortezomib at 1 nM or 3 nM did not induce significant death in myeloma cells (Figure 5C). Myeloma cells were treated with the AMP analog 5-amino-1-β-D-ribofuranosyl-imidazole-4-carboxamide, AICAR, a validated AMPK activator (Figure 5D) [30]. At the indicated concentrations AICAR also did not induce cell death. However, AICAR did increase the effect of bortezomib on autophagosomes and myeloma cell death (Figure 5E, F).

**Figure 6:**
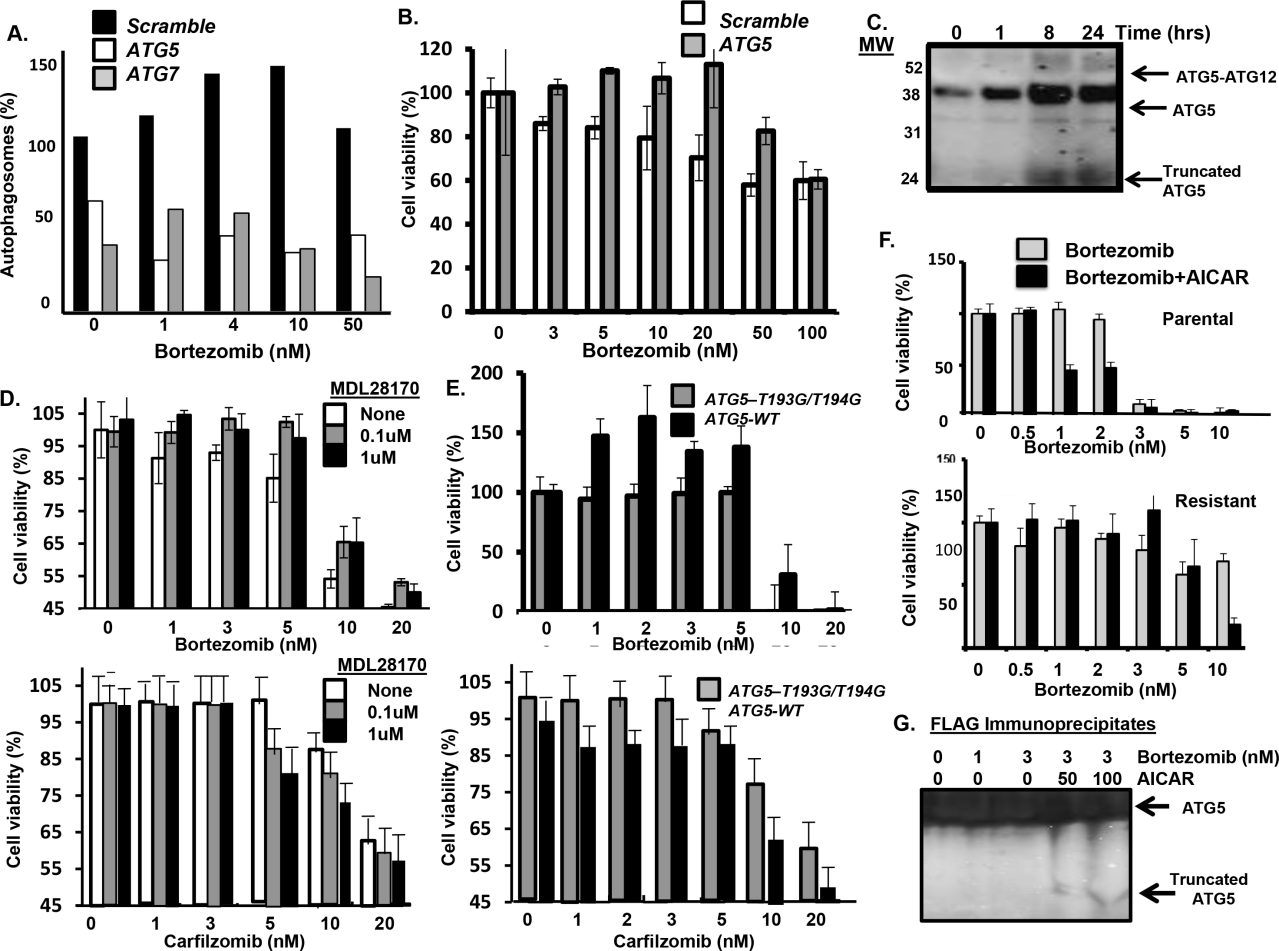
Effect of AICAR on ATG5 **(A)** Effect of *Atg5* or *Atg7* silencing in myeloma cells on bortezomib-induced autophagosome formation. **(B)** Effect of *Atg5* silencing in myeloma cells on bortezomib treatment and cell viability. Cells were treated with bortezomib at indicated concentrations and viability determined by the XTT assay. Shown are the results of triplicate measurements. Error bars represent the standard deviations. **(C)** Effect of bortezomib on ATG5 conjugation to ATG12 as well as cleavage of ATG5. Myeloma cells were treated with bortezomib for indicated times, total protein precipitated and precipitates probed using an antibody specific for ATG5. Shown is the ATG5-ATG12 conjugate (52 kDa), ATG5 (36 kDa) and truncated ATG5 form (24 kDa) detected by western blot. **(D)** Effect of the calpain inhibitor MDL28170 on myeloma cells treated with bortezomib. At the concentrations of 0.1 or 1uM, MDL28170 was not toxic to myeloma cells. Viability was determined using the XTT assay. Values represent the arithmetic mean of triplicate measurements and error bars represent the standard deviation. **(E)** Effect of the calpain-resistant ATG5 mutant on bortezomib treatment of myeloma cells. Cells were transfected with plasmids that expressed either *Atg5-WT* or an *Atg5 mutant (T193G-T194G)*. Myeloma cells were treated with bortezomib at indicated concentrations and viability was determined using the XTT assay. Values represent the arithmetic mean of triplicate measurements and error bars represent the standard deviation. **(F)** Effect of AICAR addition on parental and drug-resistant myeloma cells treated with bortezomib. Myeloma cells were treated with bortezomib at indicated concentrations and viability was determined using the XTT assay. Values represent the arithmetic mean of triplicate measurements and error bars represent the standard deviation. **(G)** Myeloma cells were transfected with a plasmid that expressed FLAG-tagged Bcl2. Cells were then treated with bortezomib, lysates prepared and immunoprecipitated using an anti-FLAG antibody. Immunoprecipitates were separated by SDS-PAGE and probed using an anti-ATG5 antibody to detect the ATG5 forms association with FLAG-tagged Bcl2.

*ATG5 cleavage promotes bortezomib-induced cell death.* Similar to CT26 cells, *Atg5* knockdown in myeloma cells also inhibited bortezomib-induced autophagosome formation and cell death (Figure 6A, B). Bortezomib promoted the covalent conjugation of ATG5 (36 kDa) to the Ub-like modifier ATG12 (~16 kDa) to form a 52 kDa product (Figure 6C). Apoptosis is triggered by the calpain-mediated cleavage of ATG5 to generate a truncated 24 kDa fragment. Calpain-mediated cleavage eliminates the autophagy-inducing activity of ATG5 but the truncated ATG5 product translocates to mitochondria to promote apoptosis [31]. Bortezomib treatment of myeloma cells generated the truncated 24 kDa form of ATG5 as seen by western blot. Myeloma cells were then treated with bortezomib in the presence or absence of the calpain inhibitor MDL28170 and the effect of viability determined (Fig. 6D). At relatively low doses, addition of the calpain inhibitor MLN28170 reduced the cytotoxic effect of bortezomib as well as carfilzomib. Myeloma cells were also transfected plasmids that expressed either *Atg5-WT* or an *Atg5-T193G-T194G*, a calpain-resistant ATG5 mutant (Figure 6E). Cells that expressed the calpain-resistant form of ATG5 were also less senstive to bortezomib- or carfilzomib-induced death. The results of these experiments support the notion that the effect is generalized to other proteasome inhibitors and not specific to bortezomib. Finally, we found that AICAR enhanced bortezomib-induced cell in both parental and drug-resistant cells and enhanced the bortezomib-induced production of the 24 kDa truncated form of ATG5 (Figure 6F, G). Calpain-mediated cleavage has been reported to eliminate the autophagosome-forming activity of ATG5 but to promote it's pro-apoptotic functions. Specifically, truncated ATG5 translocates to mitochondria and binds and antagonizes anti-apoptotic Bcl-2. Myeloma cells were transfected with a plasmid that expressed FLAG-Bcl2, treated with bortezomib and AICAR as indicated, lysates prepared and immunoprecipitated and probed with an ATG5 antibody. Bortezomib treatment alone did not lead to a significant association of truncated ATG5 with FLAG-tagged Bcl2. However, treatment with AICAR and bortezomib enhanced the association of truncated ATG5 in the FLAG-tagged Bcl2 immunoprecipitates.

## DISCUSSION

The UPS represents the major protein degradation pathway in eukaryotes and evidence points to active crosstalk with the autophagy pathway [32, 33]. Inhibition of proteasomes stimulates autophagy as a compensatory, pro-survival strategy to sequester or eliminate unwanted protein aggregates. Likewise, inhibition of autophagy compromises degradation of Ub+proteasome substrates [34]. Our results indicate that physiologically-relevant doses of bortezomib trigger AMPK+ULK-dependent induction of autophagy. Studies in yeast, *C. elegans* and higher eukaryotes demonstrate that AMPK acts as a convergence point to couple the cellular response to environmental cues, nutrient availability and stress with autophagy, growth and survival. AMPK positively regulates autophagy and our results demonstrate that AMPK is both required for bortezomib-induced autophagosome formation. Genetic knockout or chemical inhibition of AMPK reduced the effect of bortezomib at low doses to induce autophagosome formation and was consistent with prior studies that demonstrated that cellular response to stressors is context-dependent.

The induction of autophagy may have opposing effects on cell fate. Autophagy is described as a double-edged sword since under certain conditions it constitutes a pro-survival stress adaptation to suppress apoptosis. In contrast, evidence also links autophagy and many autophagy effectors to cell death pathways. Consequently, chemotherapeutics generally invoke an initial cytoprotective, autophagic response as well as cell death. Here, we show that bortezomib can trigger autophagy, apoptosis or both outcomes – dependent upon the dose and duration of tumor cell exposure. Bortezomib at low doses promoted AMPK-dependent autophagosome formation while higher drug doses or longer exposure triggered cell death. In related studies, AMPK deficiency was shown to attenuate ethanol-induced cardiac dysfunction through inhibition of autophagosome formation [35]. Similarly, administration of hydrogen peroxide (H_2_O_2_), generated under conditions of oxidative stress, to neuronal cells inhibited Akt and phosphoinositide-dependent kinase 1, activated AMPK*α* and induced apoptosis [36]. However, in those same studies, the expression of a dominant negative form of AMPK*α* or downregulation of AMPK*α*1 conferred partial resistance to H_2_O_2_-mediated cell death and inhibition of phosphorylation of S6K1 and 4E-BP1. The findings support an essential role for AMPK in triggering cell death.

Molecular and genetic lesions within tumors may impair the induction of autophagy, prevent apoptosis and promote tumor survival through metabolic adaptations that promote drug-resistance. Whether promoting cell survival or death, autophagy and apoptosis engage in a complex, poorly understood molecular crosstalk [37, 38]. Cells resistant to bortezomib undergo genetic reprogramming to not only increase AMPK activity and autophagosome formation but to also uncouple these processes from apoptosis and promote drug resistance. Our results suggest that this uncoupling is linked to reduced cleavage of ATG5 in drug-resistant cells. Bortezomib induced cell death more effectively in metabolically compromised cells to provide evidence that cellular energy status and AMPK activation are determinants of the cellular response to proteasomal inhibition. However, prolonged stimulation of autophagy leads to impaired cell proliferation and, therefore, AMPK has been directly associated with the activation of apoptosis. Since the autophagic and apoptotic response machineries share common effectors, e.g., ATG5, Beclin-1, Bim, that either link or polarize the response to stress, these adaptations can be leveraged as medically actionable molecular vulnerabilities to increase the drug specificity of cancer cells and spare normal cells.

The results presented here suggest that pharmacologics that activate AMPK and energy metabolism should enhance the anti-myeloma benefit of proteasome inhibitors. Metabolic alterations involved in cancer initiation, metastasis and drug resistance may reveal novel targets, promote pharmaceutical development and more effective cancer therapies. Reconciling the role of autophagy as a tumor-suppressive mechanism yet also able to promote tumor survival during stress is critical since many current cancer therapeutics activate autophagy. Autophagy is a validated therapeutic target in oncology and has led to multiple early phase clinical trials to evaluate hydroxychloroquine (HCQ) in combination with chemotherapy or targeted agents as second-line therapy for relapsed and/or refractory disease. However, HCQ requires nearly millimolar levels for efficacy, lacks target specificity and displays pharmacologic liabilities limit clinical advancement. The identification of novel agents to specifically target autophagy provides new opportunities for drug development since more potent agents are needed either as monotherapy or in synergistic combination. The AMPK activators D942 and troglitazone, similar to AICAR, were previously shown to inhibit the growth of myeloma cells [39]. It is conceivable that potent pharmacologic activators of AMPK (or its upstream regulators, e.g., LKB) will drive the autophagy pathway to overcome drug resistance and induce apoptosis.

Over the past two decades, protease inhibitors have emerged as a highly successful anti-cancer treatment modality [40]. However, the mechanism of action and mechanism(s) of drug resistance remain incompletely defined. Nonselective protease inhibitors are cytotoxic to leukemia and other cancer cell lines and the cytotoxicity is correlated with their potency as inhibitors of the proteasome but not as inhibitors of calpain and cathepsin. Bortezomib was shown to induce cell death with an IC_50_ as low as 5 nM in apoptosis-prone leukemia cells. Cell death was preceded by p21WAF1/CIP1 accumulation, an alternative marker of proteasome inhibition, and by cleavage of PARP and Rb proteins and nuclear fragmentation. The reintroduction of wildtype p53 into p53-null PC3 prostate carcinoma cells did not increase their sensitivity to proteasome inhibitors. Likewise, comparison of parental and p21-deficient cells demonstrated that p21WAF1/CIP1 was dispensable for proteasome inhibitor-induced cytotoxicity. The accumulation of wildtype p53 and induction of apoptosis are seen as independent markers of proteasome inhibition.

U266 MM cell clones resistant to bortezomib were generated and exhibited a similar sensitivity to various other pro-apoptotic stimuli [41]. Pan-genomic profiling of bortezomib-sensitive and resistant cells showed that the small heat shock protein HSPB8 was overexpressed in resistant cells. Finally, gain and loss of function experiment demonstrated that HSPB8 is a key factor for bortezomib resistance. In fact, HSPB8 may play a critical role in the elimination of aggregates in bortezomib-resistant cells that contributes to their enhanced survival [41]. ER stress from unfolded proteins is associated with the proliferation of pancreatic tumor cells, making the many regulatory molecules of this pathway appealing targets for therapy. The proliferation of a panel of 14 pancreatic cancer cell lines showed a dose- and time-dependent growth inhibition by IRE1α-specific inhibitors Growth inhibition was also noted using a clonogenic growth assay in soft agar, as well as a xenograft *in vivo* model of pancreatic cancer [41]. Cell cycle analysis showed that these IRE1α inhibitors caused growth arrest at either the G1 or G2/M phases and induced apoptosis. These results suggest that an IRE1α inhibitor is a novel approach for pancreatic cancers [42].

**Figure 7:**
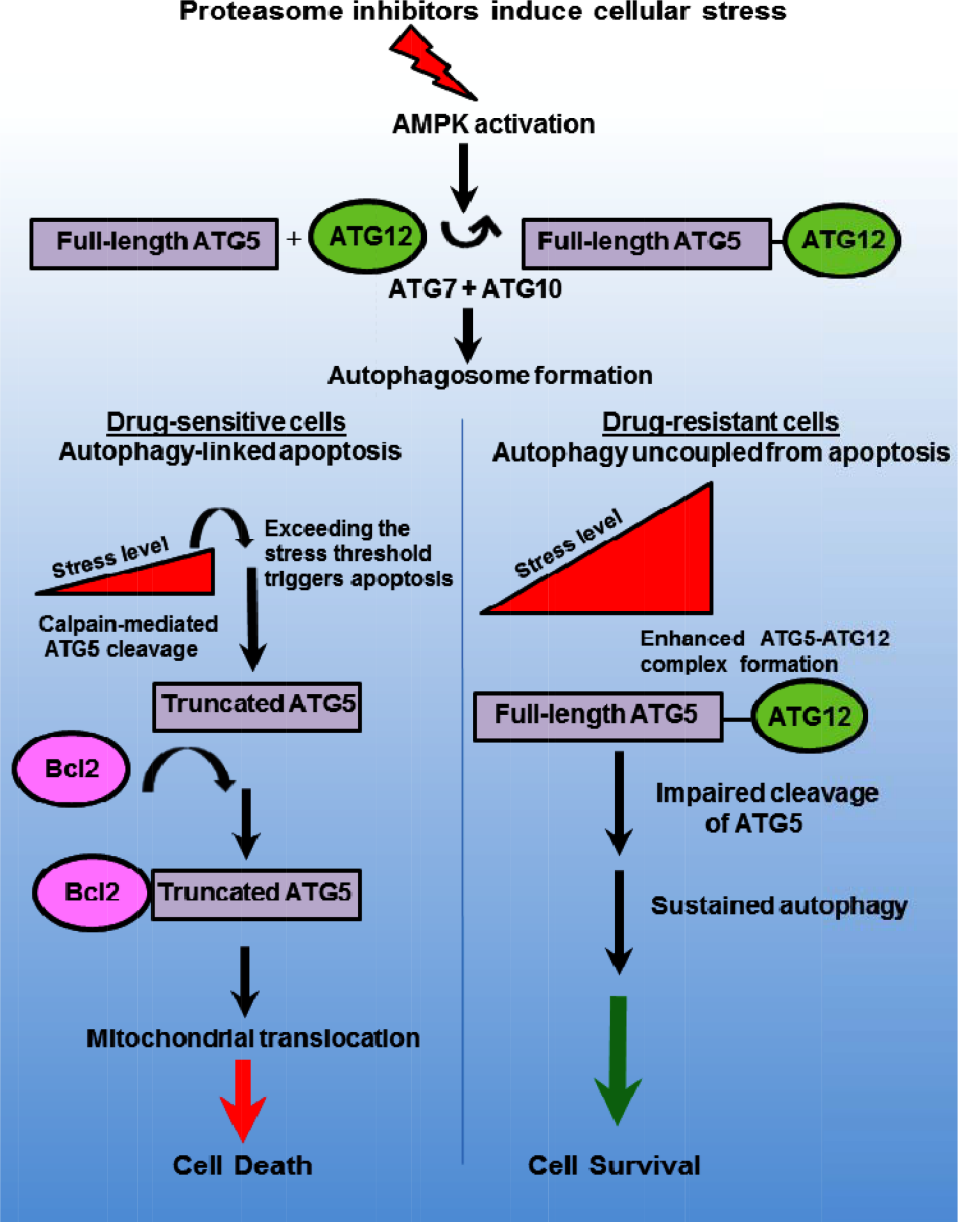
Model to depict ATG5 as a dual-functioning molecular effector in autophagy and apoptosis Proteasome inhibition promotes AMPK-dependent autophagosome formation leading to increased cellular stress. Stress increases ATG5 to ATG12 and promotes autophagosome formation and survival. The dual-functioning role ATG5 promotes autophagy but as the stress threshold is surpassed in drug sensitive cells, ATG5 is cleaved by calpain to generate a truncated form that then associates with anti-apoptotic Bcl-2. Certain anti-apoptotic proteins, e.g., Bcl2 and Bcl2A1, bind and repress pro-apoptotic proteins, e.g., NOXA and PUMA. Truncated ATG5 binding to the anti-apoptotic Bcl2 and Bcl2A1 may displaces these proteins from NOXA and PUMA. Truncated ATG5 derepresses NOXA and PUMA to promote apoptosis.

ATG5 is a dual-functioning molecular switch that promotes autophagy and apoptosis- ATG5 participates in the initial steps of autophagy through interaction with the Ub-like ATG7 and the ATG10 conjugation system (Figure 7). ATG5 is conjugated to ATG12 to form a ~52 kDa complex that binds ATG16 to structurally re-model the developing autophagophore and recruit activated LC3 to the elongating membrane. These events lead to autophagosome formation and promote cell survival. However, ATG5 is also cleaved by calpain to generate a truncated form that translocates to the mitochondria and induces mitochondrial outer membrane permeabilization (MOMP). The precise events that trigger the mitochondrial translocation of ATG5 as well as the precise link to apoptosis have not been identified. We speculate that ATG5 promotes displacement of anti-apoptotic Bcl2 family members from pro-apoptotic Bcl2 family members, e.g., NOXA and PUMA, to facilitate MOMP as a point of commitment to cell death through the mitochondrial apoptotic pathway. Other examples of dual-functioning effectors include the apoptotic caspases, beclin-1, VPS34, ATG12 [43–45] and ATG5 [29, 31, 46]. The findings that bortezomib-resistant cells exhibit a significantly greater number of autophagosomes but were insensitive to drug pointed not only to extensive crosstalk between these pathways but also that these process were functionally separable. Multiple findings reported here support the notion that ATG5 bridges these processes through distinct functional activities. Future studies are required to define the precise mechanism of ATG5-mediated apoptosis. Dual-functioning molecules such as ATG5 may constitute important convergence points between autophagy and apoptosis in the complex interplay that determines cell fate. It is reasonable to assume that they represent crucial sites to target in oncology.

## METHODS

Cell culture: RPMI8226 were from the American Tissue Culture Collection (ATCC). The N-nitroso-N-methylurethane-(NNMU)-induced, undifferentiated murine colon carcinoma cell line CT26 was transfected with scrambled siRNA or siRNA to knockdown *Atg5* or *Atg7* and provided by Laurence Zitvogel (Institut Gustave Roussy, Villejuif, France). Myeloma cells were (Gibco, Grand Island, NY), 1% pen-strep and 1% L-glutamine. All other cells were grown in DMEM/high glucose supplemented with 10% FBS, 1% pen-strep and 1% L-glutamine. MEFs were grown in complete DMEM.

Chemical reagents- Bortezomib was from ActiveBiochem (Maplewood, NJ) and other chemicals were from Sigma Chemical Co. (St. Louis, MO).

Plasmids and Transfection: Plasmids that expressed ATG5 wt or mutant forms were transfected into myeloma cells using Amaxa Nucleofector. Sequence that encoded human *Atg5* bearing a carboxy-terminal HA-tag (828 base pairs) was inserted into the vector pCI-neo. The putative calpain-cleavage site within ATG5 was predicted using the GPS-calpain cleavage detection software program [25]. Threonine residue 193 and 194 in *Atg5* were replaced by site-directed mutagenesis to encode glycine residues. Nucleotide sequencing then confirmed that the resultant vector encoded human Atg5-T193G/T194G-HA. (Genewiz, South Plainfield, NJ).

Generation of PI-resistant cells. RPMI8226 cells were exposed to successively increased concentrations of bortezomib, ixazomib or carfilzomib to generate resistant cells. Parental cells were cultured under the same algorithm in vehicle (0.5% DMSO) alone.

Real-time OCR and ECAR measurements: Time-resolved analysis of cell OCR and ECAR was obtained using the XF24 flux analyzer according to manufacturer's instructions (Seahorse Biosciences, Billerica, MA). Cells were suspended in media containing the indicated glucose or drug concentration. The plate containing the cells was then centrifuged at 40x g, the plate rotated 180^o^ and centrifugation repeated at 80 g. Media was added and the plate placed in a non-CO2 incubator for 30 minutes. The plate was then placed in the XF-24 analyzer using a 3:2:3 (mix: wait: measure) time program.

Cell Viability Determination using the XTT Assay: 5 × 10^4^ cells were plated in 96-well plates in RPMI media that lacked phenol red. Cells were then treated as indicated with drugs and incubated for 72 hours. To determine cell viability, 50 μl of activated XTT-PMS mixture (1 mg/ml XTT-20 μM PMS) was added to each well, plates further incubated for 3 hours and absorbance determined using a BMG Labtech FLUOstar OPTIMA plate reader at a wavelength of 450 nm, position delay of 0.5 seconds and 20 flashes/well.

Quantification of Autophagosomes: 1 × 10^6^ cells were plated in RPMI media, treated with drugs as indicated and autophagosomes quantitated by flow cytometry using the Enzo cyto-ID autophagy detection kit (Enzo Life Sciences (Farmingdale, NY). The assay comprehensively measures autophagosomes with negligible staining of lysosomes. Cells were pelleted, suspended cyto-ID detection reagent, incubated in dark at 37^o^C and analyzed using FL1 of a Coulter Epics XL-MCL. The assay was optimized through the identification of titratable functional moieties that allowed for minimal staining of lysosomes while exhibiting bright autolysosomes. The mean FL1 signal from an autophagy inducer with CQ increases much higher than an inducer alone, to indicate that enhanced accumulation of autophagosomes results from increased flux.

Quantification of Apoptosis: The annexin V-FITC conjugate was used with 1 × 10^6^ cells treated with drugs as indicated and incubated at 37^o^C. Cells were collected, centrifuged, washed, resuspended in 90 μl of assay buffer and 10 μl of Annexin V, FITC conjugate. Samples were analyzed in the FITC channel of a Coulter epics XL-MCL flow cytometer.

Dye-based microscopy to detect autophagy- The Cyto-ID autophagy detection kit was used to visualize autophagosomes by microscopy. 1 × 10^6^ cells in 2 ml media were plated and incubated overnight at 37^o^C. Cells were incubated in Hank's buffered saline solution (HBSS) for indicated times. After incubation, cells were collected and centrifuged at 500rpm for 5 minutes. The supernatant discarded and the pellet resuspended in assay buffer and centrifuged again. The supernatant was discarded and the pellet resuspended in 0.5 mL freshly diluted Cyto-ID detection reagent. The sample was then incubated in dark at 37^o^C for 30 minutes. The samples were then analyzed in the green channel (FL1) using a Coulter epics XL-MCL flow cytometer.

Western blotting: 1 × 10^6^ parental or drug-resistant cells were pelleted and suspended in 100 μl of CelLyticM lysis reagent (Sigma Chemical Co) placed on ice for 5 minutes, centrifuged at 3,000 rpm for 10 minutes, boiled and loaded onto 5–20% gradient gels (Wako Laboratory Chemicals, Richmond, VA). Proteins were transferred to nitrocellulose membranes, blocked with nonfat milk and incubated with primary antibodies overnight. Blots were washed and incubated with secondary antibody. Immunoreactive bands were visualized either by radiographic film or Li-COR detection method. Membrane was washed with TBS containing 0.2% Tween (TBS-T) and incubated in Western Lightning ECL Pro (GE Healthcare, Pittsburgh, PA) for 5 minutes. Images were visualized using a KODAK film and an M35A X-OMAT X-Ray Film Processor. Primary rabbit antibodies to AMPK subunits alpha-1, alpha -2, beta-1, beta-2, gamma-1, gamma-2 and gamma-3 were from Cell Signaling Technology (Danvers, MA). The primary rabbit antibody to rabbit ATG5 antibody was from Novus Biologicals) Littleton, CO. The ACC (phospho-Ser79) antibody was from Abcam (Cambridge, MA). For Li-COR visualization, membranes were blocked with Odyssey blocking buffer for 1 hour and incubated with primary antibody overnight. Membranes were then washed with PBS containing Tween-20. Li-CoR goat anti-mouse IgG or donkey anti-rabbit secondary antibody added for 60 minutes. Membranes were washed with PBS-T and visualized using a Li-Cor Odyssey Classic image detection system with integrated software.
